# Structural Basis for Potassium Inhibition of WNK Kinases

**DOI:** 10.1021/acs.biochem.5c00825

**Published:** 2026-05-26

**Authors:** Elizabeth J. Goldsmith, John M. Pleinis, Armin Wagner, Vitaliy Mykhaylyk, Radha Akella, John M. Humphreys, Haixia He, Logan Norrell, Daryl E. Morrison, Aylin R. Rodan

**Affiliations:** † Department of Biophysics, 12334 The University of Texas Southwestern Medical Center, 5323 Harry Hines Boulevard, Dallas, Texas 75390-8816, United States; ‡ 120796 Diamond Light Source, Harwell Science and Innovation Campus, Didcot OX11 0DE, U.K.; § Research Complex at Harwell, Rutherford Appleton Laboratory, Didcot OX11 0FA, U.K.; ∥ Molecular Medicine Program, 14434 University of Utah, Salt Lake City, Utah 84112, United States; ⊥ Department of Internal Medicine, Division of Nephrology & Hypertension, University of Utah, Salt Lake City, Utah 84112, United States; # Medical Service, Veterans Affairs Salt Lake City Health Care System, Salt Lake City, Utah 84143, United States

## Abstract

WNK kinases are chloride- and osmotic-stress-regulated protein kinases recently shown to be controlled by potassium. Prior studies demonstrated the direct binding of chloride and osmotic stress-related water in WNK kinase regulation. Here, we probe potassium binding and regulation of WNK kinases via crystallography coupled with mutagenic analysis of WNK kinase autophosphorylation and activity. Crystals of unphosphorylated WNK1 grown in cesium formate, a surrogate for potassium, yielded nonsulfur scattering peaks at 5.75 keV. Mutations were introduced into amino acids flanking the anomalous diffraction peaks. Mutations in WNK1/E388 and the corresponding WNK3/E314, probing a peak close to WNK1/I384, led to reduced inhibition by potassium while maintaining kinase autophosphorylation and substrate phosphorylation activity. Other peaks probed by mutagenesis either did not bear out as potassium regulatory sites or were not validated due to the inactivity of the mutants synthesized. Previously synthesized chloride- and water-binding mutants demonstrate correlated sensitivity to chloride and potassium. Potassium, chloride, and water are all WNK inhibitors that share a common mechanism binding the same low-activity asymmetric dimer of WNK1 kinase domains.

## Graphical abstract

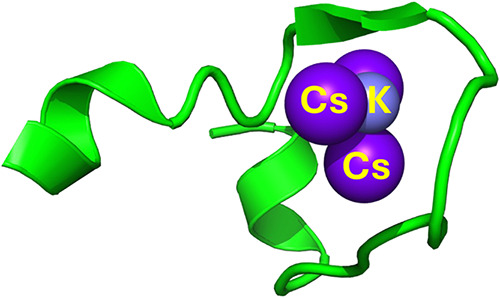

## Introduction

With No Lysine (K) (WNK) kinases are soluble intracellular serine/threonine kinases, noted for their unique constellation of catalytic residues.^1^ WNK1 and WNK4 are associated with familial hyperkalemic hypertension (Gordon’s syndrome^2^). Physiological similarities between low-potassium diets and Gordon syndrome led us to question whether potassium directly regulates WNK kinases. WNKs have the capacity to integrate multiple input and output signals.^3–5^ WNKs are regulated by chloride and by osmotic pressure.^1,6–8^ We showed recently that potassium ion inhibits mammalian WNK1, WNK3, WNK4, and *Drosophila* WNK (DmWNK).^9^ The structural basis of WNK chloride inhibition^10^ and water inhibition (in osmotic stress responses^7,11^ ) has been studied.

WNK kinases exhibit oligomerization in vitro^12,13^ and in cells.^14,15^ The regulation of WNKs studied so far fits a simple equilibrium model: the inhibitory ligands, chloride and water, bind a dimeric and inactive WNK structure.^7,10,13^ Activators, including osmotic and hydrostatic pressure, promote monomers that are autophosphorylation-competent and thus self-activating.^7,11,13^ The molecular basis for WNK osmotic activation appears to be the release of water molecule networks bound to the inactive dimer.^7^ We probed the regulatory involvement of one water network (conserved water network 1 (CWN1)) through the mutagenesis of surrounding residues. Many mutant WNKs synthesized were less active than wild type. However, mutations of WNK1/E388 and WNK3/E314 were as active as wild type, yet less sensitive to water and chloride inhibition than wild type.^7^

In addition to regulation by chloride,^10^ we have recently showed that WNK1, WNK3, WNK4, and *Drosophila* WNK are inhibited by potassium.^9^ To uncover potassium regulatory sites, we used anomalous scattering X-ray diffraction of WNK1 crystals formed in potassium formate and cesium formate. Crystallographic studies were conducted using a WNK1 mutant (WNK1/SA), eliminating the Ser382 regulatory activating phosphorylation site by mutating it to Ala, generating an inactive and unphosphorylatable dimer. Amino acids flanking crystallographic difference peaks were mutated to test changes in activity and potassium sensitivity. Bacterially expressed and purified WNK1 and WNK3 kinase domains were then used in auto- and substrate-phosphorylation assays. Further, WNK1 kinase domain and full-length WNK3 kinases immunoprecipitated from cells were assayed with the substrate SPAK.

## Materials and Methods

### Reagents, Protein Expression, and Mutagenesis of *E. coli* Expressed WNK Kinase Domains

The cloning, expression, and purification of WNK3 (118–409) (pWNK3 and uWNK3), WNK1(194–483) (pWNK1 and uWNK1), and WNK1(194–483)/S382A (WNK1/SA) were described previously.^7,10,12,16^ Many of the mutants used in this study were described previously;^7^ new mutants were produced similarly. Dephosphorylation of pWNK3 and pWNK1 to make unphosphorylated enzymes (uWNK3 and uWNK1) was also described previously^16^. The WNK phosphorylation state, as expressed in *E. coli*, was confirmed by mass spectrometry. Kinases were typically buffer exchanged into 50 mM HEPES, pH 7.4, and 150 mM NaCl for this study. A substrate GST-SPAK peptide (gSPAK, with the sequence GST-RRVPGSS*GHLHKT) was used in assays with bacterially expressed WNKs as described previously for a similar GST-peptide fusion, gOSR.^7^ The percent phosphorylation was assessed by mass spectrometry.

### In Vitro Autophosphorylation Assays of *E. coli* Purified WNK Kinase Domains

Autophosphorylation assays were conducted in general in 20 mM HEPES, pH 7.4, 20 mM MgCl_2_, 5 mM ATP, and usually in 150 mM NaCl at room temperature. Concentrations of uWNK3 or uWNK1 were 4 μM. Potassium gluconate and cesium formate concentrations were varied from 0 to 240 mM. The time scale in general was 2 h for uWNK1 and 20 min for uWNK3. Mass spectrometric analysis of activation loop peptide phosphorylation was conducted as described previously.^7^

### Crystallization of WNK1/SA in Potassium Formate and Cesium Formate

WNK1 crystals were formed using WNK1/193–493/S382A (WNK1/SA), the kinase domain of WNK1 mutated to eliminate the primary activating phosphorylation site. The original crystallization conditions contained 300 mM chloride ion, a known WNK inhibitor.^12^ New conditions were obtained by using PEG-Ion screens (Hampton Research, Inc.). Crystals were found in 20% PEG3350, in the presence of either 200 mM sodium formate or 200 mM potassium formate. Both conditions yielded 200–250 μm crystals. Similar crystals were obtained in 20% PEG3350 and 200 mM cesium formate. Multiple crystals from K^+^ and Cs^+^ conditions were cryoprotected with 20% glycerol.

### Diffraction Data Collection and Refinement

The diffraction experiments were performed at beamline *I*23 of the Diamond Light Source. The beamline is optimized for data collection at long wavelengths.^17^ Data were recorded on a semicylindrical Pilatus 12 M (Dectris AG, Switzerland) detector. The three-axis goniometer used allowed us to obtain complete data sets for the WNK1 P1 space group crystals. Diffraction at two wavelengths was measured for two crystal growth conditions. Crystals grown in potassium formate were irradiated at 3.65 keV (3.3968 Å) and 3.5 keV (3.5424 Å) (the potassium K edge is at 3.4369 Å). Crystals grown in cesium formate were irradiated at 5.75 keV (2.156 Å) and 4.95 keV (2.505 Å) (the cesium L edges are at 2.1697, 2.3134 and 2.4738 Å). Three data sets at different crystal orientations were collected over 360 deg of rotation using fine-slicing (0.10°) to obtain high-quality statistics and completeness. The crystal temperature was maintained near 80 °K. The exposure time was 0.1 s with a flux ranging from (2 to 5) × 10^10^ photons/s. Data reduction was conducted with the autoprocessing pipelines fast_DP and Xia2 [3.14.0], including Xia2_3dii and Xia2_dials. PDB entry (6CN9) and the protein sequence were provided in ISPyB to trigger the processing pipeline DIMPLE (see the Diamond Light Source Automatic Software Pipeline) to generate anomalous difference Fourier maps with Shelxc^18^ and ANODE.^19^ Data and refinement statistics are given in Table S1. The positions of anomalous peaks higher than 4.0 σ were output by ANODE from data sets both above and below the potassium and cesium edges. The top Cs^+^-formate nonsulfur peaks collected at 5.75 keV are listed in Table 1, with a complete list of cesium nonsulfur anomalous peaks presented in Table S2. Anomalous difference maps were inspected using Coot.^20^ The models and cation occupancies were refined in Phenix.^21^ Figures were drawn using PyMol (Molecular Graphics System, Version 3.0 Schrödinger, LLC.).

**Table 1:** Anomalous and |Fo-Fc| Difference Peaks

Cs^+^ anomalous difference peaks 5.75 keV (2.156 Å)		
name	chain, peak (s)	interactions
S306-adjacent	A 13, B 24	S306OH T305OH T358O
Q253-adjacent	A 21, B 7	G230O S231O Q253OE1
I384-adjacent	A 10, B 0	I384O T386O B/N216OD1 E388OE1
K^+^ anomalous difference peaks 3.65 keV (3.397 Å)		
W327-adjacent	A 4.65	V323CD1 S326OH
I384-adjacent	A 4.58	I384O B/N216OD1
A407-adjacent	A 4.50	A407O D404OD1
|Fo-Fc| K^+^ difference peaks from 0.9795 Å data		
T301-adjacent	A 9.1, B 8.6	T301OG1 D368OD1
L371-adjacent	A 8.5, B 0	L370N L371N (chloride site)
|Fo-Fc| Cs^+^ difference peaks from 0.9795 Å data		
T301-adjacent	A 5.9, B 6.5	T301OG1 D368OD1
L371-adjacent	A 8.5, B 0	L370N L371N (chloride site)
D400-adjacent	A 8.6, B 8.0	S402OG E393OE1
E419-adjacent	A 0.0, B 7.0	E419OE1 T317O
I470-adjacent	A 7.0, B 7.0	B/I470N B/H339/ND1 chloride binding?

Diffraction data from WNK1/SA crystals grown in potassium formate and cesium formate were collected at the Advanced Photon Source (APS). X-ray data were collected at beamline 19-ID, yielding diffraction to 1.8 Å resolution at wavelength 0.9795 Å. Integration and scaling were performed with the HKL-3000 software suite.^22^ Data collection and refinement statistics are given in Table S3 for reflections out to 2.0 Å, where the CC_1/2_ was above 0.5.^23^

### Generation of Plasmids for S2 Cell Expression

Construction of pAc5-HA-WNK1 (a.a. 209–483):T2A:Neo: The kinase domain of WNK1 was PCR amplified (Phusion High-Fidelity DNA polymerase, New England Biolabs #M0530) from a full-length rat WNK1.3 cDNA,^24^ and an N-terminal HA-tag was introduced, using primers D48.1 and D48.2 (all primers listed in Table S4). The PCR product was then reamplified using primers D57.1 and D53.5 to enable Gibson assembly. The plasmid backbone pAc5 STABLE2 Neo (Addgene #32426) was amplified with primers pAc5seq3rdD and D18.2. The HA-WNK1 (209–483) PCR product was added to the pAc5 plasmid backbone via Gibson assembly (NEBuilder HiFi DNA Assembly Master Mix, New England Biolabs #E2621). A stop codon was then added at the 3′-terminus of the HA-WNK1 (209–483) sequence using Q5 site-directed mutagenesis kit (New England Biolabs #E0554) and primers D21.5 and D19.6. Sequences were confirmed by Sanger sequencing for all plasmids described.

For site-directed mutagenesis of WNK1, primers were designed using NEBaseChanger (https://nebasechanger.neb.com/). pAc5-HA-WNK1 (209–483):T2A:Neo was modified by PCR (Phusion High-Fidelity DNA polymerase, New England Biolabs no. M0530) and ligated in a KLD reaction (New England Biolabs no. M0554).

Construction of pAc5-HA-WNK3/I310A: Site-directed mutagenesis of the pAc5-HA-WNK3/WT^9^ plasmid was performed using the Q5 site-directed mutagenesis kit (New England Biolabs #E0554) with primers listed in Table S4.

Construction of pAc5-HA-WNK3/E314A, pAc5-HA-WNK3/E314Q, pAc5-HA-WNK3/L295F, and pAc5-HA-WNK3/L295F/E314Q: these mutations were introduced into pAc5-HA-WNK3/WT by GenScript.

### In Vitro Kinase Assays with Immunoprecipitated WNK1 and WNK3

*Drosophila* S2-GAL4 cells were transfected with 20 μg wild-type or mutant pAc5-HA-WNK1/kinase domain or pAc5-HA-WNK3/full-length and HA-WNK proteins were immunoprecipitated as previously described.^9^ Immunoprecipitated protein was kept up to 1 week at 4 °C, but usually used within 24 h of preparation.

Each 20 μL kinase reaction contained 2.5 μL anti-HA magnetic beads (1/20th of the preparation) supplying HA-WNK1/kinase domain or HA-WNK3/full-length, 4 μM of bacterially expressed and purified GST-SPAK/D219A,^9^ 10 mM HEPES pH 8.0, 10 mM magnesium chloride, 1 mM TCEP, and 150 μM ATP. For WNK1 assays, varying concentrations of potassium chloride/*N*-methyl-d-glucamine (NMDG) gluconate were added to maintain 100 mM chloride in each reaction while varying potassium from 0 to 300 mM. For WNK3 assays, varying concentrations of potassium chloride/NMDG gluconate were used to maintain 100 mM chloride and constant osmolality (∼614 mOsm/kg, measured on a Wescor Vapro 5520 osmometer) while varying potassium from 0 mM to 300 mM. To examine the effects of chloride, varying concentrations of potassium gluconate, magnesium gluconate, magnesium chloride, and potassium chloride were used to maintain 10 mM magnesium and 100 mM potassium while varying chloride from 0 to 150 mM. NMDG gluconate was used to maintain constant osmolality (∼360 mOsm/kg measured osmolality). Reactions proceeded for 4 h at 37 °C. To quench the reaction, an equivalent volume of 2× Laemmli buffer was added, and samples were boiled for 5 min at 95 °C. Beads were then removed by magnetic immobilization, and 20 μL of supernatant was separated by 8% SDS-PAGE gel, followed by Western blotting with primary antibodies. Primary antibodies were used at 1:1,000 dilution: pSPAK (Ser373)/phospho-OSR1 (Ser325) (rabbit, Millipore No. 07-2273, lot No. 2778850) and tSPAK [mouse, GeneTex anti-STK39 (2E10) No. GTX83543, lot No. 334211]. Secondary antibodies were used at 1:10,000 dilution: goat antirabbit (Li-Cor, No. 926-32211) and antimouse (Li-Cor, No. 926-68070). Linearity for the pSPAK and tSPAK antibodies was determined by comparing 0.5x, 1x, and 2x samples.

To quantify Western blots, each tSPAK and pSPAK dominant band pixel intensity was measured using ImageJ, and background pixel intensity was subtracted. We observed some degree of SPAK/D219A phosphorylation in the absence of WNK (see Figure S5B for an example). This was measured for each preparation of purified SPAK/D219A by comparing the pSPAK/tSPAK ratio to that of WNK1 or WNK3 at 0 mM K^+^ across multiple replicates. The control on each Western (usually 0 mM potassium) pSPAK/tSPAK ratio was assigned a value of 1, and the pSPAK/tSPAK ratio for the SPAK/D219A control without WNK was subtracted. The remaining pSPAK/tSPAK ratios on the same blot were normalized to this value.

### Statistics

*R*-values (r.m.s.d of points to the lines) were calculated in DataGraph (Visual Data Tools, Inc.) with curves fit to the form *L*/(1+ exp (−*h*/*x*–*x*_o_)) where *L*, *h*, and *x*_o_ are fitted variables. Significance, where reported, was calculated using one-way ANOVA or *t* test in Excel and GraphPad Prism (* indicates α of <0.05, ** < 0.01, ***<0.001, ****<0.0001). The goodness of fit between the scattering curve and crystallographic dimer or monomer models was calculated as described.^25^ Curve fitting was carried out either in DataGraph or GraphPad Prism (GraphPad Software, Inc.).

## Results

Single-crystal diffraction from unphosphorylatable WNK1(194–483)/S382A (WNK1/SA)^12^ crystals grown in potassium or cesium formate generated anomalous and |Fo-Fc| difference maps that indicated sites of cation binding. The top peaks in the cesium anomalous data collected at the Diamond Light Source were not proven to be potassium regulatory sites. Thus, we chose to use mutagenesis to test several of the putative cation-binding sites as potassium regulatory sites. The locations of the difference peaks observed and studied here by mutagenesis are shown in Figure 1A.

**Figure 1:**
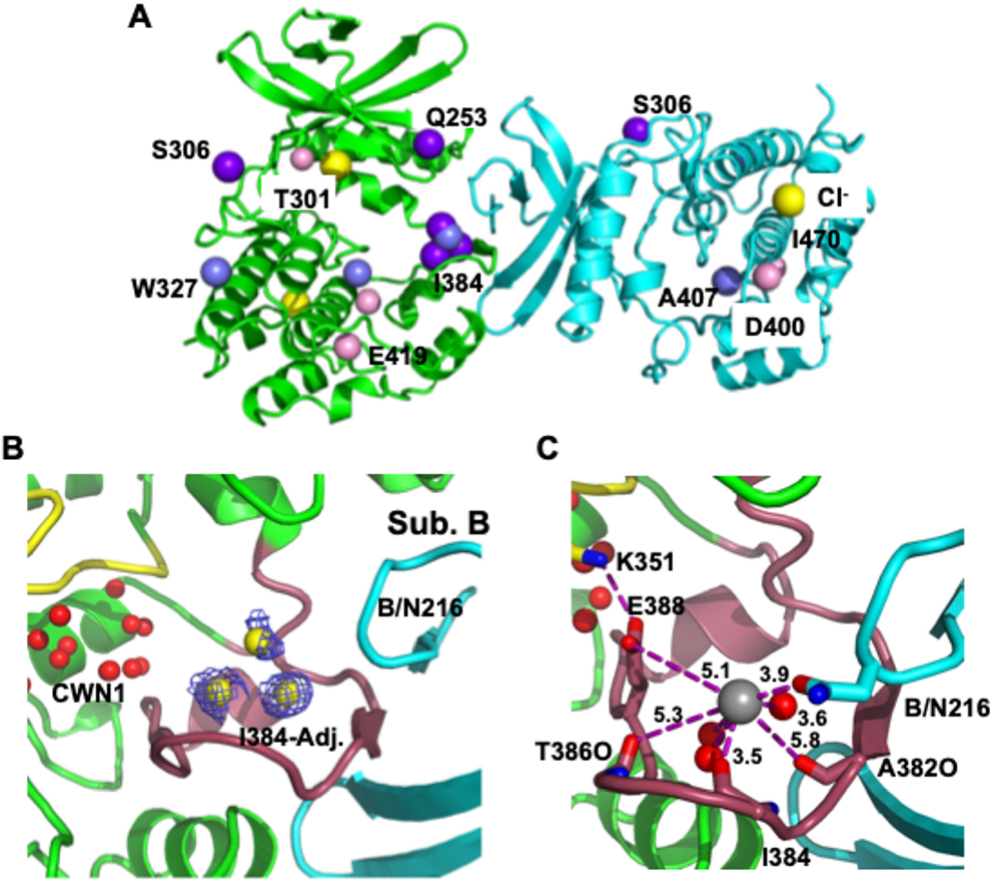
Location of anomalous difference and |Fo-Fc| peaks from WNK1/SA crystals grown in Cs^+^-formate or K^+^-formate. (A) Location of anomalous difference peaks observed in Cs^+^ (purple)- and K^+^-formate (light purple), and difference peaks observed at 0.979 Å (pink) probed in this study. The S306, A407, D400, and E419 sites were present in both chains. Subunit A, green; subunit B, cyan. Chloride ion is yellow. The chloride near T301 was published previously.^10^ The chloride near I470, present in both chains, is from this study. (B) Trio of peaks near I384 and B/N215. Cesium atoms are yellow, and anomalous electron density is blue. Waters in CWN1 are red. Activation Loop, plum, and Catalytic Loop, yellow. (C) Side chains and carbonyls of I384, T386, E388, K351, and B/N216 with distances (Å) to the potassium ion indicated (see Table S5 for cesium ion distances, which are shorter).

### Interactions of Cesium and Potassium Ions Observed by Diamond Light Source Anomalous Diffraction

Crystals of WNK1/SA grown in cesium or potassium formate gave crystals similar to those of unliganded WNK1/SA. The crystals were cryoprotected similarly, in glycerol, and diffracted to 2.2 Å (Table S1). Data collected at 5.75 keV (2.156 Å) on Cs^+^-formate-containing crystals exhibited anomalous difference peaks between 5 and 15 sigma on cysteine and methionine sulfur atoms (Table S2). The sulfur-related peaks were similarly present in data collected at 4.165 keV (2.505 Å), below the cesium L-edges (not shown). Data for uWNK1/SA crystallized in potassium formate were collected at 3.65 keV (3.3968 Å) and 3.5 keV (3.5424 Å). Three peaks between 4 and 4.6 sigma were not sulfur-associated (Table S2).

Positions of anomalous peaks in the Cs^+^-formate and K^+^- formate Diamond Light Source (DLS) data sets are shown in blue in Figure 1A. |Fo-Fc| peaks collected at 0.9795 Å tested in this study as potassium regulatory sites are shown in pink (Figure 1A). I384-adjacent peaks were observed in both the cesium and potassium anomalous scattering difference maps. The trio of peaks in the cesium map is shown in Figure 1B. The potassium anomalous peak is near the center of the cesium peaks. This site is the best validated as a potassium regulatory site by the mutagenesis assays described below (Figure 1B). WNK1/A/I384 is in the activation loop immediately following tight subunit–subunit β-sheet interactions made by residues 380 and 381. The backbone carbonyl moieties of WNK1/A residues A382, I384, and T386 are unligated and face solvent in the absence of ions. A382 is the phosphor-acceptor serine in wtWNK1. The ligation of Cs5–7 and K1 is listed in Table S5, including the distance to E388. The K-1 distances to residues ligating potassium or cesium are shown in Figure 1C. Electrostatic interactions link the cesium ion with I384O (3.1 Å) and T386O (3.4 Å) in the A-chain, with a longer interaction with N216OD1 in the B-chain (5.5 Å). Cs5 also forms a fairly short electrostatic interaction with E388 (4.2 Å). Cs7 forms interactions with I384O (3.3 Å) and A382O (4.0 Å) (Table S5); typical cesium–carbonyl oxygen distances are ∼3.3 Å.^26^ The interactions observed for K-1 are on the higher end of potassium -carbonyl O distances (3.5–4.0 Å).^27^ Both cesium and potassium induced conformational change on a distal residue, K351 (in the Catalytic Loop), where K351NZ adopts a closer contact with E388OE2 (Figure 1C).

The most intense anomalous difference peak in the Cs^+^-formate 5.75 keV map (Figure 2A,B), named S306-adjacent, neighbors the Crossover Connection and β6 on both the A and B subunits ligated by S306OH, T305OH, and T358O (Figure 2B). The second most intense anomalous peak, named Q253-adjacent, is on top of the phosphate binding ribbon (Figure 2C) and is ligated by A/G230O, S231O, and Q253OE1 (Figure 2D). ANODE refinement showed that the S305-adjacent and Q253-adjacent cesium ions are ∼40% occupied. Mutagenic analysis, however, did not support these sites as potassium regulatory sites (see below).

**Figure 2:**
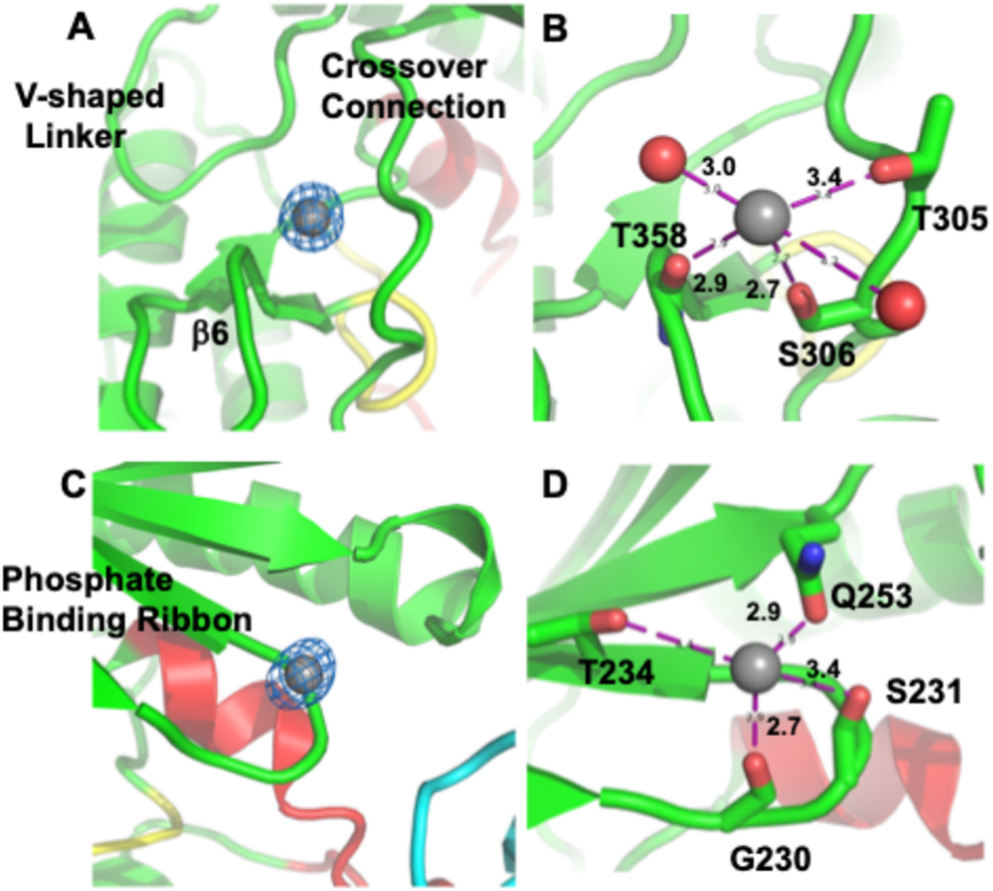
Strongest anomalous difference peaks, S306- and Q253-adjacent, from WNK1/SA crystals grown in Cs^+^-formate (5.75 keV). (A) 26 sigma peak on B chain and 15 sigma peak on A chain adjacent to WNK1/S306. (B) Same peak as in B showing the side chains of S306, T305, and the backbone carbonyl of T358. (C) 21 sigma peak at the phosphate binding ribbon showing the cartoon of uWNK1/SA (PDB file 6CN9). (D) Same as (C) showing the carbonyl oxygen atoms of G230, S231, and the side-chain of Q253. Coloring as in Figure 1B,C.

In the K^+^-formate data collected at 3.65 keV, in addition to the I384-adjacent peak noted above, two peaks, labeled A407-adjacent and W327-adjacent (Table 1), were observed in the A-chain only (Figure 1A). Neither were supported as potassium regulatory sites in the mutant assays.

### Advanced Photon Source Diffraction of WNK1/SA Crystals Grown in Potassium and Cesium Formate

Crystallographic data were also collected at APS at 0.9795 Å on WNK1/SA crystals grown in potassium formate or cesium formate. In the |Fo-Fc| map of the Cs^+^-formate uWNK1 crystals, two strong difference peaks were observed in the A subunit. The peaks are in the back of the active site between the Crossover Connection and the N-terminus of the 3/10 helix (Figure S1). One peak is near T301 (9.1 sigma); the second peak (8.5 sigma) overlaps with the chloride binding site, L371 (Figure S1B). Additional smaller difference peaks were observed in the K^+^- APS data set (Tables 1 and 2). Most of these were on the surface, making only a single contact with the protein. Peaks near A/I470N and B/I470N are flanked by nitrogen atoms, suggestive of anion binding, and were refined as such (the crystallization buffer is 150 mM in chloride ion).

**Table 2:** Mutations Probing Difference Peaks as K^+^ Regulatory Sites

mutant	peak assoc.	experiment (assay figure)	activity[Table-fn t2fn1] (% where known)	inhibited by K or Cs (% act. remaining[Table-fn t2fn1])	figure
**wtWNK1**		autophos.	95	K^+^ 5	[Fig fig3]A
			95	Cs^+^ 10	
		gSPAK	30	10	
**wtWNK3**		autophos.	95	70 (120 mM), 40 (240 mM)	[Fig fig5]C
**mutant based on 5.75 keV anom. map**					
WNK1/N216A	I384-adj.	autophos.	60	1	[Fig fig4]F
WNK1/I384A	I384-adj.	autophos.	5	yes (0.8)	[Fig fig4]A
WNK1/I384A	I384-adj.	gSPAK	12	yes (4)	[Fig fig4]B
WNK1/G385A	I384-adj.	pull-down	inactive	N.A.	[Fig fig4]E
WNK1/E388A	I384-adj.	autophos.	95	35	[Fig fig5]B
WNK3/I310A	I384-adj.	autophos.	50	10	[Fig fig4]C
fl-WNK3/I310A	I384-adj	pull-down	high	yes	[Fig fig4]D
WNK3/E314A	I384-adj.	autophos.	98	95 (120 mM), 80 ( 240 mM)	[Fig fig5]D
fl-WNK3/E314A	I384-adj.	pull-down	high	no	[Fig fig5]F
fl-WNK3/E314Q	I384-adj.	pull-down	high	no	S4B
fl-WNK1/S306Q	S306-adj.	pull-down	high	yes	[Fig fig3]C
fl-WNK1/T305Q	S306-adj.	pull-down	high	yes	[Fig fig3]C
WNK1/S306A/Q253A	Q253-adj.	autophos.	2.5	yes (0.6)	[Fig fig3]D
WNK1/S306A/Q253A	Q253-adj.	gSPAK	2	yes (0)	[Fig fig3]E
**3.65 keV anom. map**					
WNK1/V323Q	W327-adj.	pull-down	low	yes	S5A
WNK1/S326A	W327-adj.	pull-down	inactive	N.A.	S5A
WNK1/A407 V	A407-adj.	pull-down	low	N.A.	S5B
**0.9795 K |Fo-Fc| map**					
WNK1/T301A	T301-adj.	autophos.	2	yes (0)	S6A
WNK1/T301A	T301-adj.	pull-down	inactive	N.A.	
fl-WNK3/T227A	T301-adj.	pull-down	inactive	N.A.	
**0.9795 Cs |Fo-Fc| map**					
WNK1/D400A	D400-adj.	autophos.	1	0.5	
WNK1/D400N	D400-adj.	autophos.	62	1	
WNK1/E419Q	D400-adj.	autophos.	95	5	
WNK1/D400N/E419Q	D400-adj.	autophos.	95	10	S6B

1 Fixed time point, 60 min for WNK1, 10, or 12 min for WNK3. fl, full length.

Intense |Fo-Fc| difference peaks observed in the Cs^+^-formate map near D400 and E419 (Figure 1A) were of interest because the peaks are flanked by multiple carbonyl groups or negative charges, suggestive of cation binding. The same |Fo-Fc| difference peaks were observed in the Cs^+^-formate 5.75 keV data set (data not shown). The most intense anomalous peaks in the Cs 5.75 keV data set were near Ser306 and Thr305 (Figure 2A,B) in both chains and near Gln253 (Figure 2C,D) in the A chain.

### Potassium Inhibition of WNK Kinases and Mutants

Our previous work on potassium inhibition of WNKs^9^ involved two main assays. Autophosphorylation of bacterially expressed and purified WNK kinase domains was tracked by mass spectrometry. In the second assay, HA-tagged full-length WNK kinases or kinase domains were pulled down from cells and bacterially expressed substrate SPAK was Western blotted for phosphorylation. The kinase domains of WNKs 1 and 3 (see the Materials and Methods section) fully autophosphorylate in vitro and are dephosphorylated before assay.^7^ Progress curves for uWNK3 kinase domain autophosphorylation were published.^7^ Here, uWNK1 kinase domain autophosphorylation was measured in progress curves and triplicated at 60 min (Figure 3A,B (green for WNK1) and Table 2). The lag in activity concerns arises from the autophosphorylation and dedimerization.^13^ Gluconate is used as a counterion throughout to avoid the complication of chloride and other halide inhibitory effects. Potassium gluconate strongly inhibited this activity. The cesium ion is also inhibitory (Table 2). Phosphorylation of the WNK substrate, kinase-dead SPAK/D219A, by the HA-tagged WNK1 kinase domain immunoprecipitated from *Drosophila* S2 cells is also inhibited by potassium (Figure 3C).

**Figure 3:**
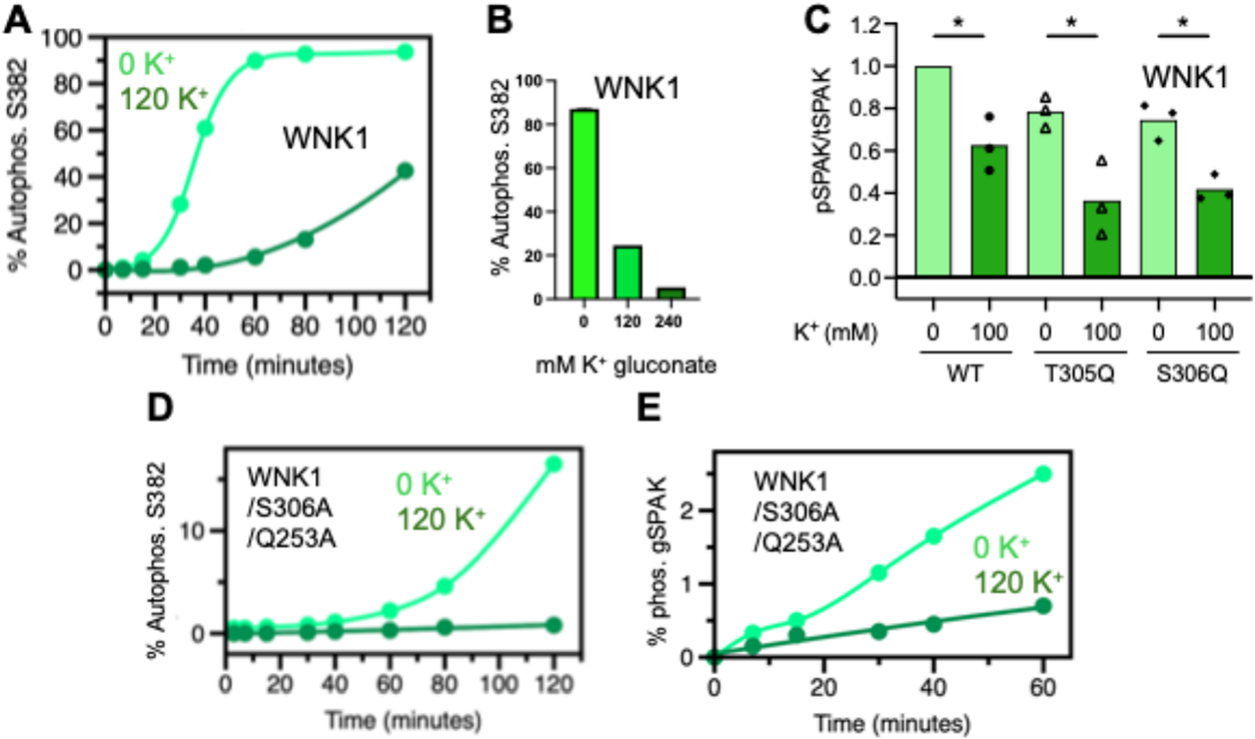
Autophosphorylation of uWNK1 and WNK1 mutant probes of the top two Cs^+^-Diamond Light Source peaks as cation regulatory sites. (A) Mass spectrometry autophosphorylation assays of wild-type (wt) uWNK1 Ser382 in 0 (light green) or 120 mM (green) K^+^-gluconate. (B) Triplicated autophosphorylation of wt uWNK1 at 60 min at the K^+^-gluconate concentrations indicated (standard error indicated). (C) Activity of HA-tagged rat WNK1 kinase domain, immunoprecipitated from S2 cells, toward bacterially expressed and purified kinase dead SPAK/D219A measured as the ratio of pSPAK/tSPAK (normalized to 1 for wtWNK1 at 0 mM potassium) for wtWNK1, WNK1/T305Q, and WNK1/S306Q. Mean with individual values shown, *n* = 3 independent experiments. *, *p* < 0.05, one-sample *t* test to a theoretical mean of 1 (WNK1/WT) or unpaired *t* test (WNK1/T305Q, WNK1/S306Q). Mass spectrometry assays of the double mutant uWNK1/S306A/Q253A for phosphorylation of (D) S382 (autophosphorylation) or (E) substrate gSPAK in the presence of potassium gluconate at the concentrations and times indicated.

To probe the validity of crystallographically observed potassium or cesium binding sites, we mutagenized nearby amino acids. Residues proximal to the most intense anomalous peaks in the Cs 5.75 keV data set, Ser306 and the adjacent Thr305 (Figure 2A,B) and Gln253 (Figure 2C,D), were mutated. HA-tagged WNK1/T305Q and WNK1/S306Q (kinase domain) were expressed in S2 cells and assayed as described above. WNK1/T305Q and WNK1/S306Q were active and maintained potassium sensitivity as measured at 0 and 100 mM K^+^ (Figures 3C and S2). Apparently, the S306-adjacent site is unlikely to be a potassium regulatory site. The Q253-adjacent site was probed in the context of a double mutant, WNK1/S306A/Q253A, measuring in vitro autophosphorylation (Figure 3D). This mutant exhibited potassium sensitivity, despite low activity. Substrate gSPAK (GST-tagged SPAK peptide containing the S-motif serine phosphorylated by WNKs,^28^) in vitro phosphorylation was tracked by mass spectrometry over time. Under the conditions used (see the Materials and Methods section), wild-type WNK1 phosphorylates 30% of gSPAK in 60 min (Table 1). In contrast, WNK1/S306A/Q253A had 10-fold less activity but remained potassium sensitive (Figure 3E). Apparently, the Q253-adjacent cesium binding site is not a potassium regulatory site.

Mutants were then synthesized to probe the significance of the WNK1/I384-adjacent anomalous difference peaks (Figure 1B,C). The initial mutants tested probed residues most proximal to the peaks, namely, I384 in WNK1 and the corresponding I310 in WNK3, and G385 and N216 in WNK1. WNK1/I384A autophosphorylation assays (Figure 4A) showed reduced activity relative to wild-type while maintaining potassium sensitivity. Similarly, WNK1/I384A gSPAK phosphorylation (Figure 4B) was also less than wild-type, whereas potassium sensitivity was maintained. The corresponding mutant in WNK3, WNK3/I310A, was less active than wild-type WNK3 in autophosphorylation assays, as described previously^7^ and as shown below, but maintained potassium sensitivity (Figure 4C) (blue for WNK3 assays). Full-length WNK3/I310A immunoprecipitated from S2 cells was also active and retained potassium sensitivity (Figure 4D). In contrast, mutation of the residue, WNK1/G385, neighboring WNK1/I384, resulted in an inactive kinase (Figure 4E). The WNK1 mutant, WNK1/N216A, autophosphorylates like wild-type and also maintains potassium sensitivity (Figure 4F). Further data, presented below, suggest that the I384-adjacent site is a potassium regulatory site. It is possible that the WNK1/I384A potassium sensitivity arises from the limited influence of the side chain that points toward solvent. Why WNK1/N216Q retains potassium sensitivity is unclear.

**Figure 4:**
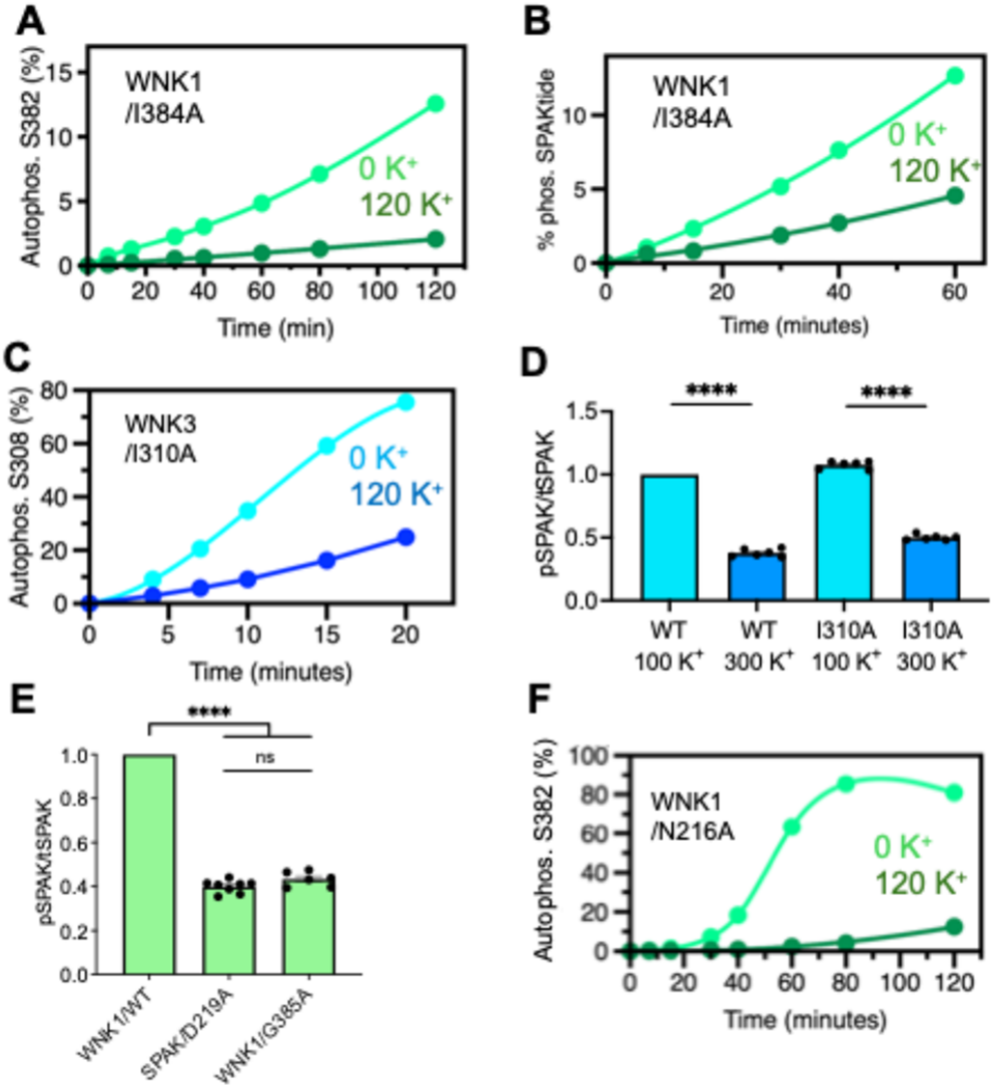
Probes of the three 10 sigma peaks in the 5.75 keV and 6 sigma peak in the 3.65 keV maps, I385-adjacent, as indicative of a cation regulatory site. Bacterially expressed WNK1/I384 in 0 (light green) and 120 (dark green) mM K^+^-gluconate in (A) autophosphorylation of Ser382 and (B) phosphorylation of substrate gSPAK. (C) In vitro autophosphorylation of WNK3 Ser308 by WNK3/I310A (homologous to WNK1/I384A) in 0 (light blue) and 120 (dark blue) mM K^+^-gluconate. (D) Activity of HA-tagged full-length human wt (wild-type) WNK3 or WNK3/I310A immunoprecipitated from S2 cells toward SPAK/D219A. The pSPAK/tSPAK ratio at 100 and 300 mM K^+^ was normalized to WNK3/WT at 100 mM K^+^, at which maximal WNK3 activity is observed in this assay.^9^ Mean ± SEM, *n* = 6. ****, *p* < 0.0001, one-sample *t* test vs theoretical mean of 1 (WNK3/WT) or unpaired *t* test (WNK3/I310A). (E) WNK1/G385A (kinase domain) assayed as in (D) in 0 mM K^+^, showing no activity above the SPAK/D219A no WNK added control, normalized to WNK1/WT at 0 mM K^+^. Mean ± SEM, *n* = 6–8. ****, *p* < 0.0001, one-sample *t* test vs theoretical mean of 1. ns, not significant, unpaired *t* test. (F) Autophosphorylation assays of WNK1/N216A in 0 (light green) and 120 (dark green) mM K^+^-gluconate over 120 min.

A further test of the I384-adjacent cesium anomalous difference peak was made by evaluating the influence of the nearby residue, WNK1/E388, and the corresponding WNK3/E314 (see Figure 1C). The mutants WNK1/E388A and WNK3/E314A gave the phenotype expected for a potassium-binding residue: high activity with loss of potassium sensitivity. Mutation of these residues was previously shown to reduce chloride and water inhibition of autophosphorylation of the WNK1 kinase domain.^7^ Here we confirm that the WNK3/E314 mutants have diminished (WNK3/E314A) or abolished (WNK3/E314Q) chloride sensitivity in the context of full-length WNK3 (Figure S3). uWNK1/E388A gave an autophosphorylation progress curve similar to (or slightly faster than) wtWNK1 in 0 mM potassium (Figure 5A,B). In 120 mM potassium, the mutant maintained more activity (Figure 5B) than wtWNK1 (Figure 5A). For example, at the 60 min time point, WNK1/E388A was ∼35% phosphorylated, whereas wtWNK1 was only ∼5% phosphorylated. wtWNK3 autophosphorylation measured in vitro is inhibited by potassium (Figure 5C), as shown previously.^9^ WNK3/E314A is more active than wtWNK3, becoming fully phosphorylated in ∼5 min. WNK3/E314A is less sensitive to potassium than wild-type: at 10 min, WNK3/E314A is fully phosphorylated even at 240 mM K^+^-gluconate, whereas wtWNK3 is only 75% and 40% phosphorylated in 120 and 240 mM K^+^-gluconate, respectively (Figure 5C,D). The exact binding constants for potassium gluconate were not measured.

**Figure 5:**
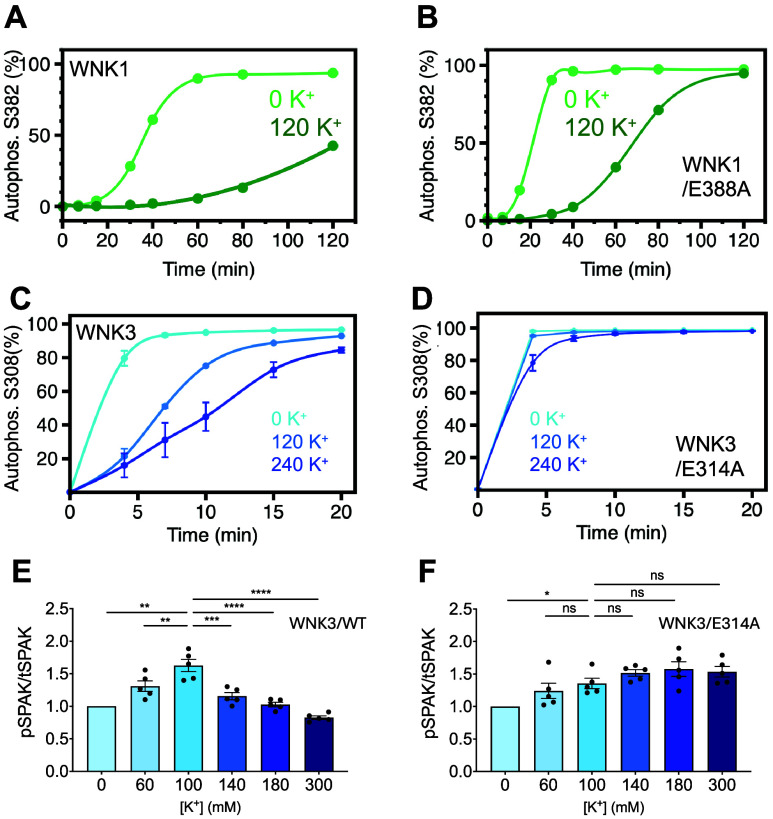
Effects of potassium on WNK1/E388A and WNK3/E314A. Autophosphorylation assays of WNK1 Ser382 in 0 and 120 mM K^+^-gluconate of (A) wtWNK1 and (B) WNK1/E388A (light green, 0 mM and dark green,120 mM K^+^-gluconate). Similar assays of WNK3 Ser308 phosphorylation in 0, 120, or 240 mM K^+^-gluconate (ascending shades of blue) for (C) wtWNK3 and (D) WNK3/E314A. Standard deviations of triplicated data (error bars). (E) HA-tagged human full-length wild-type WNK3 or (F) full-length WNK3/E314A pulled down from S2 cells. Activity toward SPAK/D219A measured as the ratio of pSPAK/tSPAK, normalized to 0 mM K^+^, in indicated ascending concentrations of K^+^. Mean ± SEM, *n* = 5. One-way ANOVA *p* < 0.0001 (E) and *p* = 0.0756 (F). ns, not significant; *, *p* < 0.05; **, *p* < 0.01; ***, *p* < 0.001; ****, *p* < 0.0001, one-sample *t* test vs theoretical mean of 1 (comparisons to 0 mM K^+^) or one-way ANOVA with Dunnett’s multiple comparisons test (comparisons to 100 mM K^+^).

We further tested these mutants in the context of the full-length enzyme, WNK3 immunoprecipitated from cells, as a function of the potassium concentration. As previously observed,^9^ WNK3 activity in this assay shows a bifurcated response to potassium, with maximal activity at 100 mM K^+^ (physiological intracellular K^+^ concentration) and inhibition at higher K^+^ concentrations (Figure 5E). In contrast, inhibition of WNK3 activity at concentrations higher than 100 mM K^+^ was not observed for WNK3/E314A (Figure 5F) or WNK3/E314Q (Figure S4). From these data, we conclude that the I384-adjacent anomalous peaks in the Cs^+^-formate Diamond Light Source data sets are sites of regulatory potassium binding.

Other peaks observed by anomalous scattering or in |Fo-Fc| maps were probed by mutagenesis. None of these mutants demonstrated the activities expected for a potassium-regulatory site. However, these data serve as negative controls validating the I384 adjacent site. Two peaks labeled W327-adjacent and A407-adjacent (Table 1) were observed in the K^+^-formate 3.65 keV data set. Both are in the active site and thus are potential regulatory sites (Figure 1A). Mutant WNK1 kinase domains, WNK1/V323Q and WNK1/S326A, were used to probe the W327-adjacent site in pull-down assays. These mutants were either of very low activity or inactive (Figure S5A). The low activity of WNK1/V323Q was inhibited by potassium (Figure S5A). The mutant WNK1/A407 V had very low activity (Figure S5B), such that the A407-adjacent site cannot be ruled out as a cation regulatory site.

Two strong |Fo-Fc| difference peaks appeared in 0.9795 Å data collected from crystals grown in both K^+^-formate and Cs^+^-formate (Figure S1). One peak is near WNK1/T301 (labeled T301-adjacent). The other is close by, near WNK1/L371 (L371-adjacent) in the active site. Previous mutational analysis of the L371-adjacent peak demonstrated reduced chloride sensitivity.^10^ The WNK1/T301A as expressed in bacteria exhibits very low activity, although maintaining potassium sensitivity (Figure S6A). The corresponding mutants WNK1/T301A and full-length WNK3/T227A immunoprecipitated from S2 cells were also inactive (Table 2). Thus, the results are inconclusive whether the T301-adjacent site is a potassium regulatory site.

Additional significant |Fo-Fc| peaks appeared in the Cs^+^-formate 0.9795 Å data (Table 1) that were present in 5.75 keV Cs^+^-formate |Fo-Fc| data (not shown). The mutants WNK1/D400N, WNK1/E419Q, and the double mutant WNK1/D400N/E419Q were synthesized and assayed (Table 2). WNK1/D400A was inactive. The three mutants, WNK1/D400N, WNK1/E419Q, and WNK1/D400N/E419Q, were fully active and potassium inhibited (data for the double mutant shown in Figure S6B). These two sites, D400-adjacent and E419Q-adjacent, are unlikely to be potassium regulatory sites.

### Co-Regulatory Phenomena in WNKs

How is potassium coregulated with other ions and water? In wild-type WNK3, chloride and potassium inhibition were additive as assayed in the *Drosophila* renal tubule.^9^ Here, we measured WNK3 (kinase domain) autophosphorylation progress curves (Figure S7A). 120 mM potassium ion has similar inhibitory effects in 50 mM chloride (black vs green), 150 mM chloride (green vs yellow), and 250 mM chloride (blue vs magenta) curves. Triplicated 12 min autophosphorylation assays similarly exhibited additivity of potassium and chloride inhibition (Figure S7B; pink vs purple and red vs dark purple).

As discussed above, WNK1/E388 and WNK1/E314 mutants have correlated decreases in chloride and potassium sensitivities. To test the generality of correlation between chloride and potassium sensitivity, previously synthesized mutants probing the role of the CWN1 in water inhibition and chloride sensitivity^7^ were tested for potassium inhibition. WNK1/K236A had modestly decreased chloride inhibition compared to wild-type^7^ and a similar decrease in potassium inhibition (Figure 6A). WNK1/K307A had similar chloride sensitivity to wild-type and also similar potassium sensitivity (Figure 6B). WNK1/D279N, WNK3/Y346F, and WNK1/M301A have increased sensitivity to both chloride^7^ and potassium (Figure 6C-E). Chloride-insensitive mutants were previously identified based on the chloride binding site, WNK1/L369F, and the corresponding WNK3/L295F.^10^ Full-length WNK3/L295F was tested for potassium effects and exhibited reduced inhibition (Figure S8). This is consistent again with coupling of potassium and chloride regulation. This coregulation is discussed below.

**Figure 6:**
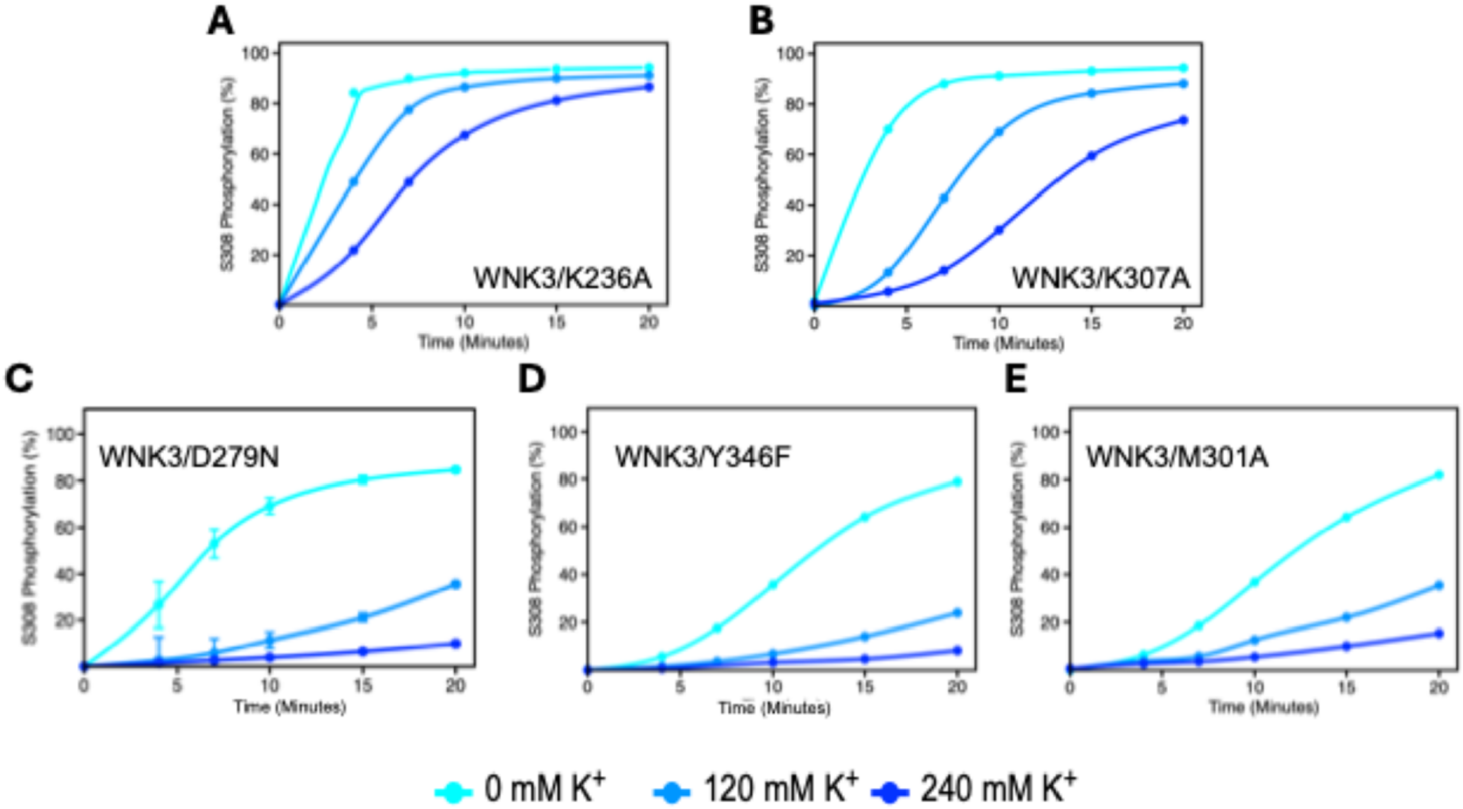
Basal autophosphorylation activity and effects of potassium on uWNK3 CWN1 mutants. (A) uWNK3/K236A, (B) uWNK3/K307A, (C) uWNK3/D279N, (D) uWNK3/Y346F, (E) uWNK3/M301A. Ser308 autophosphorylation measured with 4 μM uWNK3, 30 °C at K^+^ gluconate concentrations indicated. Bars indicate standard error from triplicate independent experiments.

## Discussion

Prior data demonstrated that potassium is an inhibitor of multiple full-length WNKs in cells and kinase domains in vitro.^9^ Here we sought to determine potassium binding sites in the WNK kinase domain. Anomalous difference signals from cesium binding to uWNK1 supported the idea that potassium binds near WNK1/S306 and WNK1/Q253. However, mutagenic analysis ruled out these sites. The I384-adjacent site was observed in anomalous difference maps from crystals grown in both potassium formate and cesium formate. Mutation of the nearby residue WNK1/E388 and the corresponding residue in WNK3, WNK3/E314, yielded enzymes with activity slightly greater than wild-type in autophosphorylation assays, together with reduced potassium sensitivity. It is interesting that there is only a single peak in the potassium anomalous map and 3 peaks in the cesium anomalous map. Perhaps the cesium atom is too large to adopt the same ligation as the potassium ion. The position of the potassium peak suggests that the potassium ion is ligated directly by the backbone carbonyl of I384 and the B-chain N216 side-chain oxygen. Potassium binding sites to proteins are variable, with observations of 3 to 8 ligand sites.^27^ Involvement of backbone carbonyls is a hallmark of potassium binding sites.^27^ Potassium works on WNKs within the physiological range (∼120 mM), a weak interaction. The intracellular potassium concentration is quite high (∼100–120 mM).^29^ The sparse physical interactions are consistent with a necessarily weak binding constant. The fact that mutations of contact residues in the I384-adjacent site is also interesting. Apparently, the side-chains, facing away from the binding site, do not strongly influence binding.

Our data do not rule out the possibility of additional potassium regulatory sites. A metal binding site near T301 remains a possibility since |Fo-Fc| peaks are observed there. However, mutation of WNK1/T301 or the corresponding WNK3/T277 yielded an inactive enzyme, so we were unable to assess these mutants for potassium regulation.

Prior observations implicate a dimer-to-monomer transition in the activation of WNKs, an inactive dimer, and an autophosphorylation-competent monomer. Small-angle X-ray scattering, static light scattering, NMR,^13^ and crystallography^7^ support this model, with inhibitors chloride and water binding the inactive dimer, and osmotic and hydrostatic pressure inducing the monomer. The autocatalytic progress curves for WNK autophosphorylation with or without potassium (for example, Figure 3A) fit the dimer–monomer mechanism, as suggested previously with respect to chloride regulation.^7^ It is also interesting that all three WNK inhibitors, chloride, potassium, and water, bind this inactive asymmetric dimer.

The present data leave several questions to be addressed in future studies. Specifically, how does potassium bind and regulate phosphorylated forms, are there multiple potassium-binding sites, and how do WNK isoforms compare in their potassium sensitivity?

Potassium inhibits autophosphorylation and may inhibit the activity. The crystallographic data studied here were collected on an inactive configuration using the mutant WNK1/S382A. Future work will address how potassium binds to the active phosphorylated form of WNKs and whether the serine hydroxyl of S382, the primary activating phosphorylation site, is directly involved in potassium binding to the wild-type inactive form.

## Conclusions

Potassium is an inhibitory ion of WNK kinases and directly binds to an inactive dimeric configuration, as demonstrated here for WNK1. Several ion-binding sites were observed by anomalous scattering from cesium and potassium ions, present in excess. Mutation of a ligating amino acid (E388 in WNK1) to an ion bound adjacent to the subunit interface exhibited the characteristics of a potassium inhibition site, maintaining high activity and reduced potassium sensitivity. Other binding sites probed by mutagenesis either could not be evaluated for potassium inhibition due to low activity or did not exhibit these characteristics and thus are unlikely to be potassium regulatory sites.

## Supplementary Material



## Notes

X-ray data and refinement statistics, Diamond Light Source anomalous difference peaks, primers, figures showing electron density, chloride sensitivity, potassium sensitivity of selected mutants, interactions at the I384-adjacent site (PDF)
