# Supplementary material for: Structural Basis for Potassium Inhibition of WNK Kinases
Source: Biochemistry. 2026 May 26;65(12):1922–31. doi: 10.1021/acs.biochem.5c00825 (PMC13397483; doi:10.1021/acs.biochem.5c00825)
Supplement: Supplementary file 1 [file bi5c00825_si_001.pdf]

SUPPORTING INFORMATION

Structural basis for potassium inhibition of WNK Kinases

Elizabeth J. Goldsmith[a*], John M. Pleinis[d], Armin Wagner [b,c], Vitaliy Mykhaylyk[b], Radha Akella[a], John M. Humphreys[a], Haixia He[a], Logan Norrell[d], Daryl E. Morrison[d], and Aylin R. Rodan[d,e,f,*]

[a]Department of Biophysics, The University of Texas Southwestern Medical Center, 5323 Harry Hines Boulevard, Dallas, TX 75390-8816, USA

[b]Diamond Light Source, Harwell Science and Innovation Campus, Didcot OX11 0DE, United Kingdom

[c]Research Complex at Harwell, Rutherford Appleton Laboratory, Didcot OX11 0FA, United Kingdom

[d]Molecular Medicine Program, University of Utah, Salt Lake City, UT 84112, USA

[e]Department of Internal Medicine, Division of Nephrology & Hypertension, University of Utah, Salt Lake City, UT 84112, USA

[f]Medical Service, Veterans Affairs Salt Lake City Health Care System, Salt Lake City, UT 84143, USA

Corresponding author
*Elizabeth J. Goldsmith: elizabeth.goldsmith@utsouthwestern.edu;
*Aylin R. Rodan: aylin.rodan@hsc.utah.edu

Table S1. Diamond Light Source datasets and refinement
Table S2. Non-sulfur anomalous difference peaks in the Diamond Light Source data
Table S3. Advanced Photon Source data on crystals grown in K^+-formate and Cs^+-Formate: crystallographic data and refinement
Table S4. Primers
Table S5. Potassium and cesium ion interactions at the I384-adjacent site

Supplemental Figure S1. |Fo-Fc| peaks from uWNK1/SA crystals grown in K^+-formate (0.9795 Å).
Supplemental Figure S2. Potassium sensitivity of WNK1/T305 and WNK1/S306 mutants.
Supplemental Figure S3. Chloride sensitivity of S2-cell expressed WNK3, WNK3/E314A, and WNK3/E314Q.
Supplemental Figure S4. Potassium sensitivity of WNK3/E314 mutants.
Supplemental Figure S5. Mutational analysis of peaks observed in the 3.65 keV anomalous difference map in the presence of K^+-formate, W327- and A407-adjacent.

Supplemental Figure S6. T301-adjacent and D400/E419-adjacent difference peaks in 0.9795 Å data probed by mutagenesis.
Supplemental Figure S7. Autophosphorylation of uWNK3 Ser308 by potassium and chloride.
Supplemental Figure S8. Potassium sensitivity of WNK3/L295F.

Table S1. Diamond Light Source datasets and refinement

	uWNK1/SA + K^+-formate	uWNK1/SA + Cs^+-formate
**Diffraction data**		
Energy (keV/ Wavelength (keV,Å)	3.65, 3.397	5.75, 2.156
Resolution	33.-2.2	38.6-2.2
Completeness	86.4 (53.9)	99.3 (90.2)
Multiplicity	5.0 (2.3)	4.7(3.7)
I/σ(I)	6.8 (0.7)	32.5 (10.6)
Rmerge (I)	0.183 (1.190)	0.053 (0.119)
Rmerge (I+/-)	0.128 (0.939)	0.026 (0.083)
$CC_{1/2}$	0.977 (0.316)	0.998 (0.975)
Wilson B-factor	37.06	28.20
Anomalous completeness	79.6 (44.2)	88.6 (75.7)
Anomalous multiplicity	2.6 (1.2)	2.4 (2.0)
Anomalous correlation	0.538 (0.212)	0.81 (0.55)
dF/F	0.190	0.066
dI/σ(dI)	1.4	2.4
Total observations	121117 (1732)	131094 (4825)
Unique observations	24252 (769)	28029 (1294)
Space group	P1	P1
Unit cell dimensions (Å)	*a,b,c* = 38.42, 57.61, 65.58	*a,b,c* =38.6,57.83,65.52
Angles (°)	α,β,γ = 89.0,89.7,89.3	α,β,γ= 89.1,89.3,89.4
**Structure**		
Rwork/Rfree[b] (last shell)	0.17/0.22	0.18/0.24(0.21/0.25)
Non-H protein atoms	4677	4663
Waters	197	220
RMSD in bond length (Å)[c]	0.007	0.007
RMSD in bond angles(°)[c]	1.4	1.4
Average B-values ($Å^2$)	36	24
Ramachandran plot stats. (%)		
Most favored region	99.0	99.0
Disallowed region	1.3	1.0
Molprobity Score	1.3	2.3

Residues missing from the model	194-209, 482-483	194-209, 482-483
R.M.S.D. to 6CN9 (Å)	0.34	0.37

Table S2. Non-sulfur anomalous difference peaks in the Diamond Light Source data

Peak Name	**DLS Name**	***Peak Height***	***Occupancy***	***B-Factor***
**Cs^{+}-Formate 5.75 keV**				
B/S306-Adjacent	Cs1	26.23	0.43	62.66
A/Q253-Ajacent	Cs2	23.11	0.38	63.97
A/S306-Adjacent	Cs3	15.08	0.57	89.9
A/G274-Adjacent	Cs4	13.67	0.37	76.38
A/I384-Adjacent A	Cs5	10.95	0.4	85.88
A/I384-Adjacent B	Cs6	6.89	0.2	114.5
A/I384-Adjacent C	Cs7	7.87	0.38	83.02
B/E270-Adjacent	Cs8	12.82	0.41	67.23
B/D465-Adjacent	Cs9	11.36	0.36	82.79
A/T436-Adjacent	Cs10	10.69	0.49	95.33
A/S437-Adjacent	Cs11	6	0.39	94.96
A/E270-Adjacent	Cs12	7.61	0.51	99.02
A/N216-Adjacent	Cs13	7.83	0.45	81.4
**K^{+}-Formate 3.65 keV**				
A/I384-Adjacent	K1	6.37	Low#	N.D.
A/W327-Adjacent	K2	4.33	Low	N.D.
A/A407-Adjacent	K3	4.50	Low	N.D.

#Due to the low magnitude of anomalous signal, the refinement yields very low occupancies (<0.1).

Table S3. Advanced Photon Source data on crystals grown in K^+-formate and Cs^+-Formate: crystallographic data and refinement

	**uWNK1/SA + K^+-formate**	**uWNK1/SA+Cs^+-formate**
Space group	P1	P1
Unit cell dimensions (Å)	*a,b,c*=38.25, 57.72, 65.61	*a,b,c* =38.43,57.81,65.64
Angles (°)	α,β,γ=89.0,89.6,89.2	α,β,γ = 91.1,90.4,89.6
Wavelength (Å)	0.9795	0.9795
Resolution (Å)	43.7-1.98 (1.98-2.04)	43.0-1.99 (1.99-2.04)
Unique reflections (last shell)	34524 (2350)	28373 (805)
Completeness(%) (last shell)	91 (83)	77 (32)
I/σ (last shell)	-	
Rsym, Rpim (last shell)[a]	0.11, 0.06 (0.72, 0.45)	0.08, 0.04, (0.25, 0.12)
Redundancy (last shell)	3.9 (3.3)	5.5 (5.0)
CC1/2 (last shell)	0.53	1.0 (0.96)
Wilson B factor	32.1	
**Structure**		
Rwork/Rfree[b] (last shell)	0.18/0.23 (0.23/0.29)	0.19/0.24(0.22/0.29)
Non-H protein atoms	4677	4663
Waters	247	244
RMSD in bond length (Å)[c]	0.007	0.007
RMSD in bond angles(°)[c]	0.91	1.4
Average B-values ($Å^2$)	36.2	24
Ramachandran plot stats. (%)		
Most favored region	95.9	99.0
Allowed region	3.9	1.0
Molprobity Score	5.25	2.3
Missing residues	194-209, 482-483	194-209, 482-483
R.M.S.D. to 6CN9	0.205	0.23

[a] $R_{sym} = \sum | I_{avg} - I_j | / \sum I_j$.
[b] $R_{factor} = \sum | F_o - F_c | / \sum F_o$, where F_o and F_c are observed and calculated structure factors, respectively, R_{free} was calculated from a randomly chosen 5% of reflections excluded from the refinement, and R_{factor} was calculated from the remaining 95% of reflections.
[c] r.m.s.d is the root-mean-square deviation from ideal geometry.

Table S4. Primers

Plasmid	Forward primer (5' – 3')	Reverse primer (5' – 3')
WNK1/WT: D48.1/D48.2	ATGTACCCATACGATGTTCCAGATTACGCTCAGGAGGAAAGGAACCAG	ATGTACCCATACGATGTTCCAGATTACGCTACCAAGGCAGTGGGAATG
WNK1/WT: D57.1/D53.5	CCTACTAGTCCAGTGTGGTGGAATTCGCCACCATGTACCCATACGATGTTCCAGATT	CCGCATGTTAGAAGACTTCCTCTGCCCTCAAGTGTTTCCTCCTGGAAAAAGGC
pAc5: pAc5seq3rdD/D18.2	CTTGAGGGCAGAGGAAGTCTTCTAACATGC	GGTGGCGAATTCCACCAC
WNK1/WT: D21.5/D19.6	TGACTTGAGGGCAGAGGAAGT	TGTTTCCTCCTGGAAAAAG
WNK1/A407V	ACGGATTCATCGTACTTC	TGATGTTTATGTGTTTGGGATGTGCATG
WNK1/G385A	GAGTGTGATAGCCACCCCAGAGTTTATGG	TTGGCAAAAGAAGCCCGC
WNK1/T305Q	TGAACTAATGCAGTCTGGAACACTTAAAACG	GTCACTAAAACAATGCATTTTTTC
WNK1/S306Q	ACTAATGACACAGGGAACACTTAAAACGTAC	TCAGTCACTAAAACAATGC
WNK1/V323Q	GAAAATCAAACAGTTAAGAAGCTGGTGTCGGC	ATCACTTTAAACCTTTTTAAGTAC
WNK1/S326A	AGTTTTAAGAGCCTGGTGTCGGC	TTGATTTTCATCACTTTAAACC
WNK3/I310A	TAAGAGTGTCGCTGGAACTCCTGAGTTTATG	GCAAATGAGGTACGCATTAAG

Table S5. Potassium and cesium ion Interactions at the I384-adjacent site

Ion	Valence	Ligands				
K-DLS 9ZPN		I384O	B216ND1	E388OE1	Wat	
K-1	4	3.5	3.9	5.1	3.6 (E98O)	
Cs-DLS 9ZCK					A382O	T386O
Cs-5	4	3.1	5.5	4.2	5.6	3.4
Cs-6	weak	5.5	5.5	5.7	7.3	6.8
Cs-7	4	4.0	4.4	6.3	3.3	5.9
		T305OD1	S306OD1	T358O	Wat	
Cs-1 (B-chain)	4	3.2	2.9	3.3	3.4 (E381O)	
Cs-3 (A-chain)	4	3.4	3.2	3.1	3.4 (E432O)	
		G230O	S231O	Q253OE1		
Cs-2 (A-chain)	3	2.9	3.6	3.2		

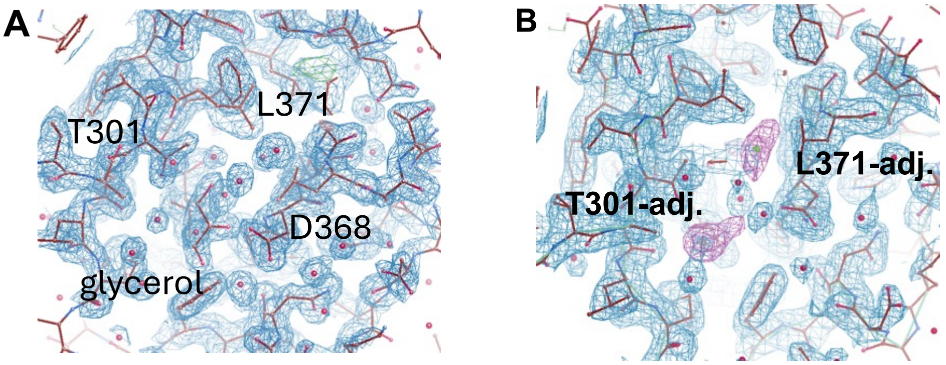

Supplemental Figure S1. |Fo-Fc| peaks from uWNK1/SA crystals grown in K^+-formate (0.9795 Å). (A) Electron density in the active site of WNK1/SA in the absence of potassium (WNK1 coordinates from PDB 6CN9). (B) 10 sigma peak on A-chain of uWNK1/SA in potassium formate. The peaks are adjacent to T301, D368 (catalytic aspartic acid), and L371 (implicated in chloride binding (Piala, *et al,* 2014)).

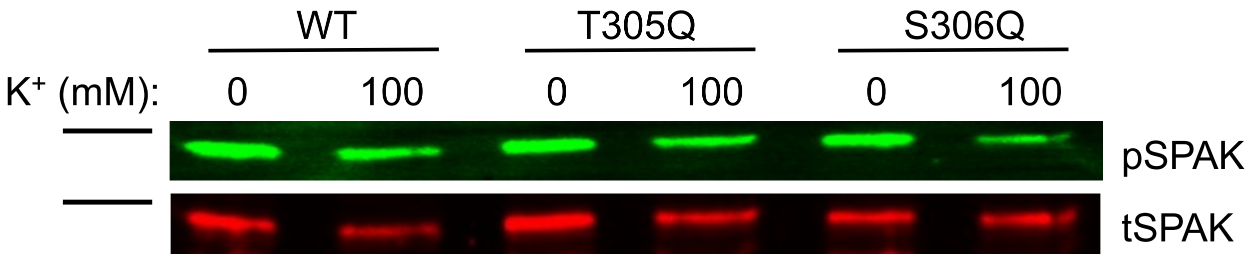

Supplemental Figure S2. Potassium sensitivity of WNK1/T305 and WNK1/S306 mutants. Assays of HA-tagged WNK1 kinase domain immunoprecipitated from S2 cells toward SPAK/D219A at 0 vs. 100 mM K^+. Western blotting was performed using antibodies to phosphorylated (pSPAK) and total (tSPAK) SPAK. The pSPAK/tSPAK ratio reflects WNK activity. Molecular weight marker, 75 kDa.

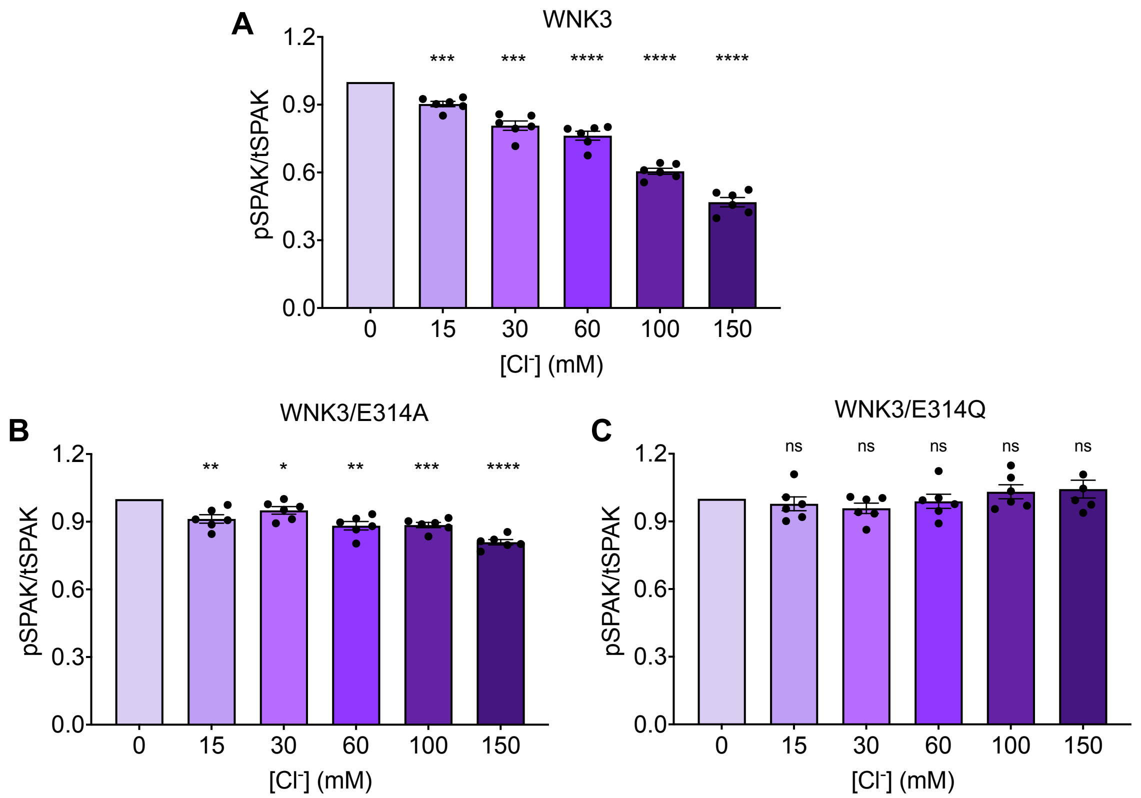

Supplemental Figure S3. Chloride sensitivity of S2-cell expressed WNK3, WNK3/E314A, and WNK3/E314Q. Assays of HA-tagged full-length human (A) wtWNK3, **(**B) WNK3/E314A, or (C) WNK3/E314Q immunoprecipitated from S2 cells toward SPAK/D219A measured as the ratio of pSPAK/tSPAK in indicated ascending concentrations of chloride ion (see Methods). The pSPAK/tSPAK ratio was normalized to the ratio obtained at 0 mM Cl^-. Mean±SEM with individual data points shown, n=6. *, $p<0.05$; **, $p<0.01$; ***, $p<0.001$; ****, $p<0.0001$, one sample t-test to a theoretical mean of 1.

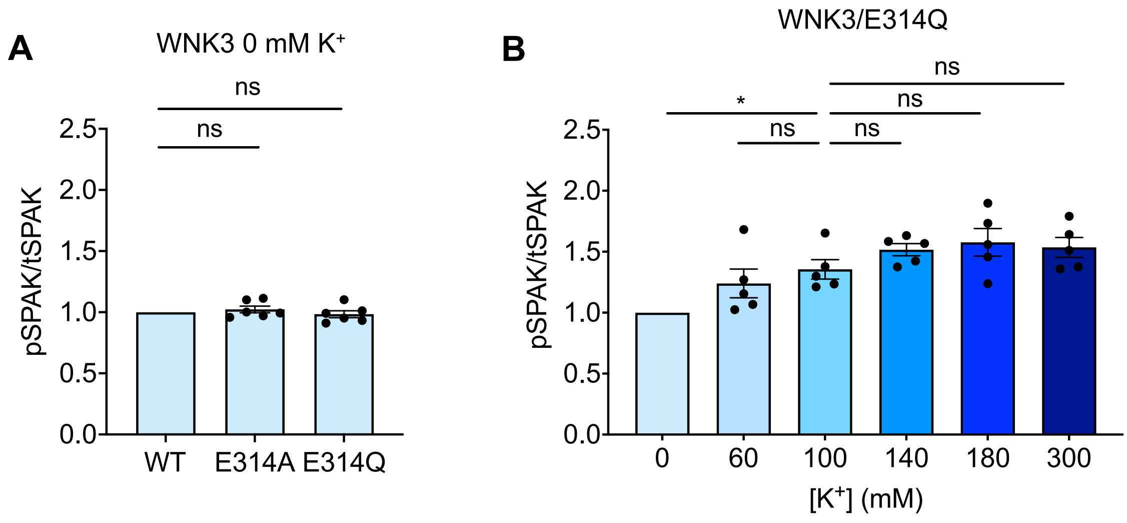

Supplemental Figure S4. Potassium sensitivity of WNK3/E314 mutants. A) Baseline activity of immunoprecipitated HA-tagged full-length human WNK3, WNK3/E314A and WNK3/E314Q, toward SPAK/D219A at 0 mM K^+. The pSPAK/tSPAK ratio was normalized to the ratio obtained with WT WNK3. Mean±SEM with individual data points shown, n=6. ns, not significant, one sample t-test to a theoretical mean of 1. B) Activity of immunoprecipitated HA-tagged full-length human WNK3/E314Q toward SPAK/D219A in indicated ascending concentrations of potassium ion. The pSPAK/tSPAK ratio was normalized to the ratio obtained at 0 mM K^+. *, $p<0.05$, one sample t-test to a theoretical mean of 1. ns, not significant, one-way ANOVA ($p=0.0639$) with Dunnett's multiple comparisons testing.

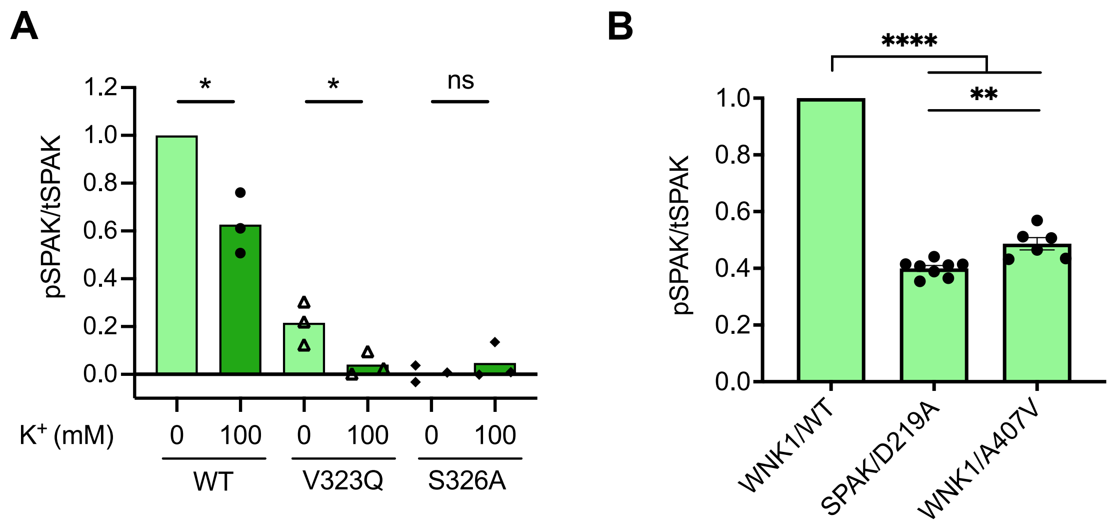

Supplemental Figure S5. Mutational analysis of peaks observed in the 3.65 keV anomalous difference map in the presence of K^+-formate, W327- and A407-adjacent. (A) Mutational probe of the W327-adjacent peak. Kinase domains of wtWNK1, WNK1/V323Q and WNK1/S326A assayed as in Figure 3C (0 and100 mM K^+), with pSPAK/tSPAK ratios normalized to wtWNK1 at 0 mM K^+. Mean with individual data points shown, n=3. *, $p<0.05$, one sample t-test to a theoretical mean of 1 (WNK1/WT) or unpaired t-test (WNK1/V323Q and WNK1/S326A). (B) Similar assays of WNK1/A407V in 0 mM K^* revealing low activity. ****, $p<0.0001$, one sample t-test to a theoretical mean of 1. **, $p<0.01$, unpaired t-test comparing SPAK/D219A control (without WNK) to WNK1/A407V.

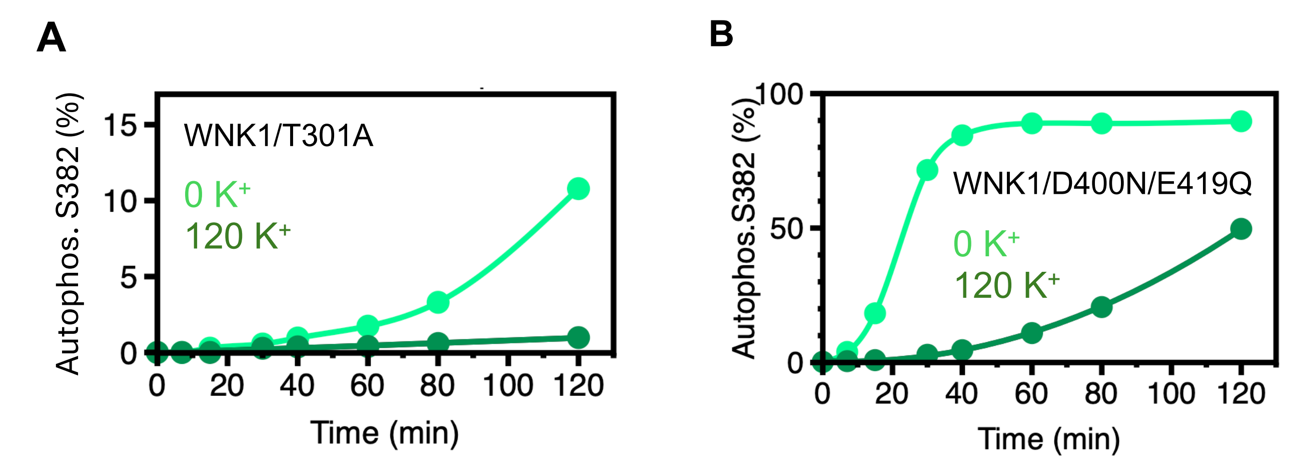

Supplemental Figure S6. T301-adjacent and D400/E419-adjacent difference peaks in 0.9795 Å data probed by mutagenesis. (A) Autophosphorylation assay of T301A. Light green, 0 mM K^+-formate, dark green, 120 mM K formate. (B) Autophosphorylation of the Cs^+-APS |Fo-Fc| map inspired double mutant D400N/E419Q measured in 50 mM chloride ion, 0 and 120 mM K^+-formate.

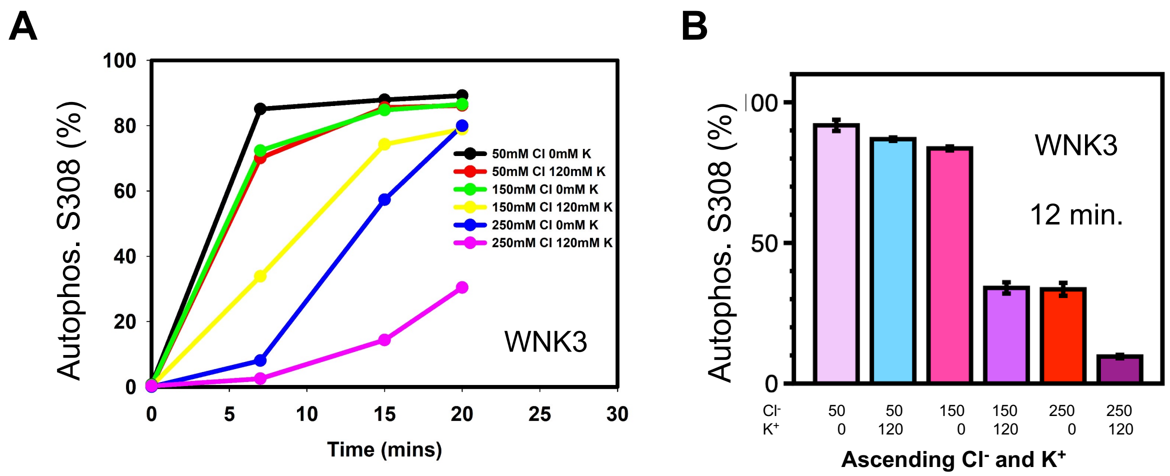

Supplemental Figure S7. Autophosphorylation of uWNK3 Ser308 by potassium and chloride. (A) Incorporation of phosphate into uWNK3 Ser308 was measured by mass spectrometry at the indicated concentrations of chloride and potassium ion. (B) Triplicated measurements of potassium gluconate inhibition of uWNK3 Ser308 autophosphorylation at 60 minutes (standard errors indicated).

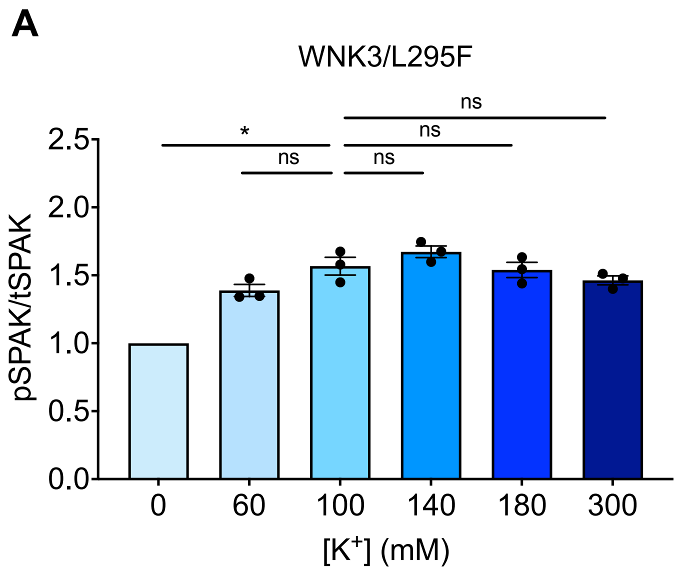

Supplemental Figure S8. Potassium sensitivity of WNK3/L295F. Activity of immunoprecipitated HA-tagged full-length human WNK3/L295F toward SPAK/D219A in indicated ascending concentrations of potassium ion. The pSPAK/tSPAK ratio was normalized to the ratio obtained at 0 mM K^+. *, $p<0.05$, one sample t-test to a theoretical mean of 1. One-way ANOVA, $p=0.0212$. ns, not significant, Dunnett's multiple comparisons testing.
